# ELABELA protects against diabetic kidney disease by activating high glucose-inhibited renal tubular autophagy

**DOI:** 10.7555/JBR.37.20220214

**Published:** 2023-11-15

**Authors:** Xiyin Zheng, Lulu Yin, Jing Song, Juan Chen, Wensha Gu, Min Shi, Hong Zhang

**Affiliations:** Department of Endocrinology, the Affiliated Huai'an No. 1 People's Hospital of Nanjing Medical University, Huai'an, Jiangsu 223300, China

**Keywords:** diabetic kidney disease, ELABELA, renal injury, fibrosis, renal tubular autophagy

## Abstract

ELABELA (ELA), an endogenous ligand of the apelin receptor (also known as apelin peptide jejunum [APJ]), has been shown to decrease in the plasma of patients with diabetic kidney disease (DKD). In the current study, we explored the potential function as well as the underlying mechanisms of ELA in DKD. We first found that the ELA levels were decreased in the kidneys of DKD mice. Then, we found that ELA administration mitigated renal damage and downregulated the expression of fibronectin, collagen Ⅳ, and transforming growth factor-β1 in the *db/db* mice and the high glucose cultured HK-2 cells. Furthermore, the autophagy markers, Beclin-1 and LC3-Ⅱ/LC3-Ⅰ ratio, were significantly impaired in DKD, but the ELA treatment reversed these alterations. Mechanistically, the inhibitory effects of ELA on the secretion of fibrosis-associated proteins in high glucose conditions were blocked by pretreatment with 3-methyladenine (an autophagy inhibitor). In summary, these *in vivo* and *in vitro* results demonstrate that ELA effectively protects against DKD by activating high glucose-inhibited renal tubular autophagy, potentially serving as a novel therapeutic candidate for DKD.

## Introduction

Diabetes, an endocrine system disease characterized by abnormally high blood glucose levels, is among the most prevalent and rapidly increasing diseases worldwide and is projected to affect 783.2 million adults by 2045^[1–2]^. Diabetic kidney disease (DKD), a major microvascular complication resulting from long-standing hyperglycemia through various mechanisms, is a primary cause of end-stage renal disease^[2–3]^. Traditional DKD treatments have primarily focused on lifestyle interventions, hyperglycemia and hypertension control strategies, and the use of renin-angiotensin system blockades^[4]^. However, these approaches are not always feasible or effective. Approximately 40% of DKD patients progress to an end-stage renal disease, necessitating dialysis and/or transplantation^[5–6]^, thereby imposing a substantial economic burden.

DKD is characterized by the accumulation of extracellular matrix (ECM), hypertrophy, and fibrosis in kidney glomerular and tubular cells^[7–8]^. While previous studies predominantly investigated the association between glomerular injury and DKD, some recent evidence has suggested that renal tubular injury plays a crucial role in the development of DKD^[9–11]^. In early DKD stages, the increased tubule reabsorption initiates or exacerbates hyperfiltration, ultimately resulting in diabetic kidney damage^[12–13]^. This nephron region, particularly the metabolically active proximal tubular segment, is susceptible to various renal insults^[14]^. Renal tubule damage contributes to abnormal accumulation of ECM, culminating in irreversible renal function deterioration and renal tubulointerstitial fibrosis^[15–16]^. Consequently, comprehending the mechanisms underlying renal tubular injury is vital for global DKD prevention and control.

Autophagy, a conserved process responsible for the removal of superfluous or damaged organelles, is essential for maintaining renal homeostasis^[17]^. The critical role of autophagy in renal tubular cell function has been extensively documented in prior studies, wherein the selective deletion of *Atg5* or *Atg7* in proximal tubules led to progressive kidney damage and premature kidney aging^[18]^. Autophagy dysfunction has also been observed in kidney proximal tubules in DKD. For example, defective autophagy was reported in proximal tubular epithelial cells in various DKD animal models and kidney biopsies of DKD patients, highlighting its significance in DKD development^[19–20]^; moreover, autophagy deficiency in proximal tubules exaggerates renal hypertrophy and tissue damage in DKD mice^[21]^, suggesting that impaired autophagy in proximal tubular cells may be crucial in promoting the progression of DKD. These findings render the enhancement of renal tubular autophagy activity a potential therapeutic target for DKD.

ELABELA (ELA), a peptide discovered in 2013, serves as the second endogenous ligand of the G protein-coupled receptor apelin peptide jejunum, in addition to apelin^[22–23]^. Given its high conservation between species, ELA is believed to perform fundamental physiological functions^[24]^. ELA has been demonstrated to regulate cardiac functions, promote angiogenesis, relax aortic blood vessels, maintain water homeostasis, *etc*.^[25–26]^. Exogenous ELA administration alleviates proteinuria and hypertension (pre-eclampsia symptoms) in the ELA knockout pregnant mice^[27]^. Furthermore, ELA has been reported to ameliorate renal injury in mice after ischemia/reperfusion by enhancing autophagy^[28]^. At present, there are few studies have investigated the role of ELA in DKD, but our previous research identified a close association between ELA and DKD, that is, the serum ELA levels were reduced in DKD patients, with a negative association between ELA levels and DKD deterioration^[29]^. However, potential effects and mechanisms of ELA in DKD remain unclear.

In the current study, we evaluated therapeutic effects of ELA on DKD both *in vivo* (the *db/db* mice) and *in vitro* (high glucose-exposed HK-2 cells) models. Additionally, we investigated whether the ELA treatment might protect against DKD by activating renal tubular autophagy.

## Materials and methods

### Animals and pharmacological intervention

Sixteen male *db/db* mice (C57BL/KsJ) with type 2 diabetes mellitus and eight normal littermates (*db/m*, 7-week-old) were obtained from the Model Animal Research Center at Nanjing University, China. The mice were housed in a specific pathogen-free animal facility under controlled temperature 22 (± 2) ℃, humidity of 55% (± 5%), and photoperiod (12 h light/dark cycle), with *ad libitum* access to a standard diet and water throughout the experimental period. The *db/db* mice were randomly divided into two groups: the *db/db* group (*n* = 8, receiving an equal volume of saline solution) and the *db/db* + ELA group [*n* = 8, treated with ELA-21 at 5 mg/(kg·day) for 8 weeks]. Age-matched *db/m* mice served as the normal control group and received saline administration concurrently. ELA-21 peptide (98% purity), with the sequence LRKHNCLQRRCMPLHSRVPFP, was procured from GenScript (Nanjing, Jiangsu, China). All drugs were administered intraperitoneally once daily at 10:00 a.m. Blood glucose levels and body weight were monitored weekly during the experiment. To acquire urine samples, all mice were individually kept in metabolic cages (TSE Phenomaster, Thuringia, Germany) at 16 weeks of age. The urinary albumin/creatinine ratio (UACR) was detected by immunoassay (DCA 2000 system; Siemens AG, Berlin, Germany). At the end of the experiments, the mice were euthanized, and blood samples were collected. Glycosylated hemoglobin A1c (HbA1c) levels were assessed using an ultrasensitive mouse HbA1c assay kit (Crystal Chem, Illinois, USA) following the manufacturer's instructions. All animal experiments adhered to the guidelines for the Care and Use of Animals published by the Institutional Ethics Committee of Animal Care at Nanjing Medical University (Approved number: IACUC-1710010).

### Kidney histopathology and immunohistochemistry analysis

Kidney tissues were harvested immediately after euthanasia. One-half of each kidney was fixed in 4% formaldehyde, embedded in paraffin, and sectioned into 5 μm-thick slices. Then, the sections were stained with hematoxylin and eosin (H&E) and Masson's trichrome. Renal histopathology was examined under a microscope. To evaluate ELA expression in the kidneys, immunohistochemistry (IHC) staining was conducted. Following standard deparaffinization, hydration, and antigen retrieval, the paraffin sections were washed with PBS, blocked with 2% BSA, and incubated with the primary antibody (ELA, 1∶1000 dilution; Cat. #H-007-19, Phoenix Pharmaceuticals, San Francisco, CA, USA) overnight at 4 ℃. After washing three times with PBS, the sections were incubated with the corresponding secondary antibody conjugated with horseradish peroxidase. Diaminobenzidine was employed for color reaction, followed by a 10-second hematoxylin, and the tablets were sealed after natural air drying. The staining intensity was observed under an optical microscope.

### Cell culture and treatments

HK-2 cells (CRL-2190; ATCC, Manassas, VA, USA) were cultured in Dulbecco's Modified Eagle Medium/Nutrient Mixture F-12 (DMEM/F-12; Gibco, Grand Island, NY, USA) supplemented with 10% fetal bovine serum and 1% penicillin-streptomycin at 37 ℃ in a humidified atmosphere containing 5% CO_2_. HK-2 cells were exposed to normal (5.5 mmol/L) or high (30 mmol/L) glucose treatment upon reaching 50% confluence and incubated for 48 h. Subsequently, HK-2 cells in a high glucose medium were treated with or without 1 μmol/L ELA-21 peptides for 1 h. To examine whether ELA could activate renal tubular autophagy, 3 mmol/L 3-MA (an inhibitor of autophagy; Sigma-Aldrich, St. Louis, MO, USA) was pretreated for 1 h in the high glucose medium supplemented with ELA-21 peptides. Cells were then harvested with the lysis buffer for Western blotting analysis.

### Western blotting analysis

Freshly collected kidney or cultured cells were homogenized in the ice-cold RIPA buffer (Beyotime Biotechnology, Shanghai, China). The supernatant was collected by centrifuging at 12000 *g* at 4 ℃ for 20 min, and the protein concentrations were quantitated by the BCA Protein Assay Kit (Thermo Fisher Scientific, Waltham, MA, USA). The protein (50 μg) from each sample was separated by SDS-PAGE and transferred onto PVDF membranes (0.22 μm pore; Millipore, Boston, MA, USA) for immune detection. Primary antibodies against ELA (1∶1000; Cat. #ab61637, Abcam, Cambridge, UK), collagen Ⅳ (Col-Ⅳ; 1∶1000; Cat. #ab6586, Abcam), fibronectin (FN; 1∶1000; Cat. #SC-8422, Santa Cruz Biotechnology, Santa Cruz, CA, USA), transforming growth factor-β1 (TGF-β1; 1∶1000; Cat. #3711; Cell Signaling Technology, Boston, MA, USA), Beclin-1 (1∶1000; Cat. #3495, Cell Signaling Technology), microtubule-associated protein 1 light chain-3B (LC3B; 1∶1000; Cat. #L7543, Sigma-Aldrich), and GAPDH (1∶2000; Cat. #2118S, Cell Signaling Technology) were used in the current study. Images were captured with ECL prime reagent (Millipore) using the ChemiDoc Touch Imaging System (Bio-Rad, Hercules, CA, USA). For densitometric analysis, pixel intensity was quantified using the ImageJ software.

### Statistical analysis

Data were expressed as the mean ± standard deviation, and the statistical significance of differences between two groups was calculated by Student's *t*-test. Repeated measures ANOVA with Tukey's post hoc analysis was used to analyze the differences of blood glucose or body weight and the three groups as well as time. One-way ANOVA analysis followed by Tukey's multiple comparisons test was performed to analyze data more than two groups. Analyses were conducted by using SPSS version 26.0 or GraphPad Prism version 9.0, and differences were considered statistically significant with *P* < 0.05.

## Results

### ELA expression was decreased in the kidneys of the *db/db* mice

**Figure 1:**
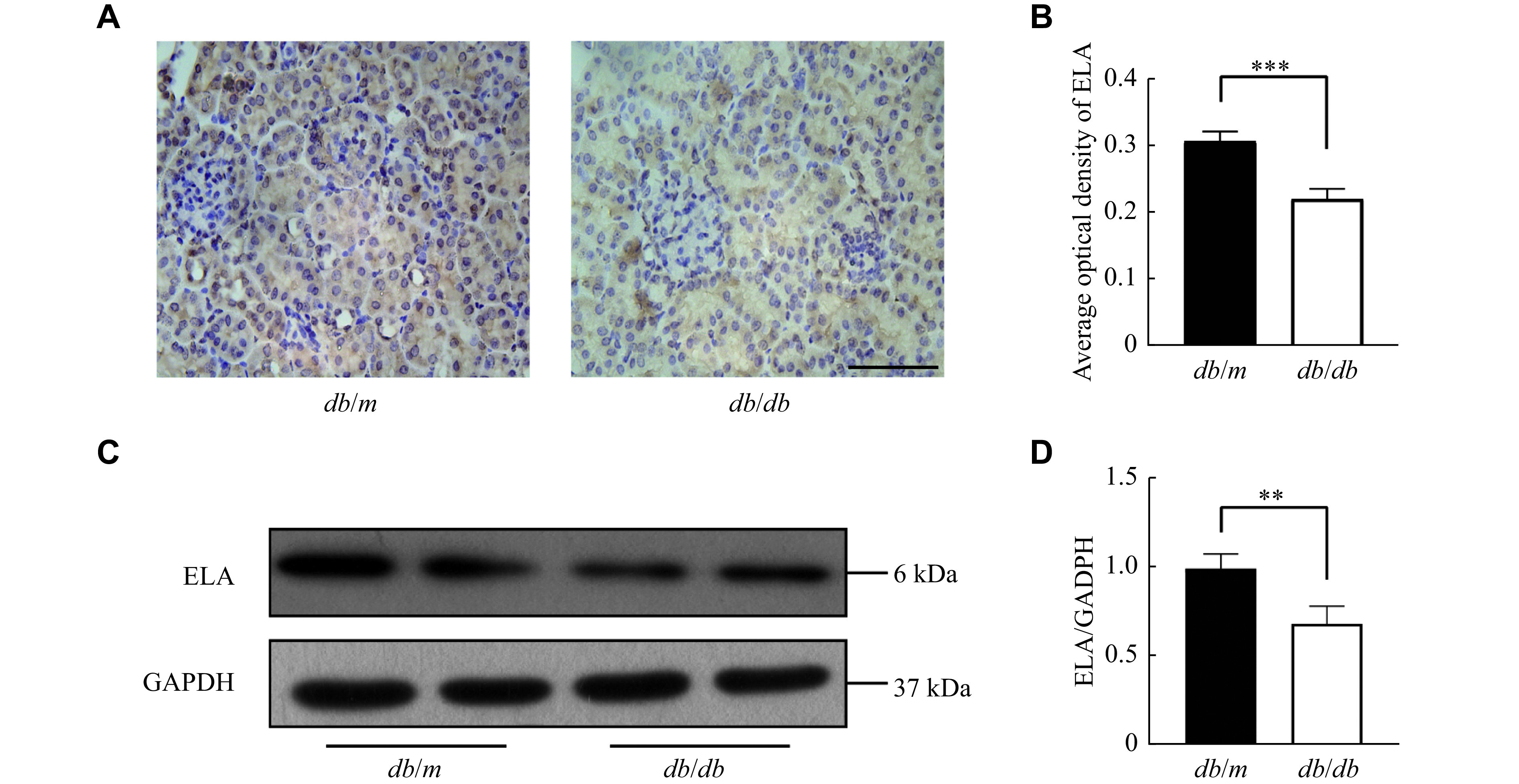
ELA expression was decreased in the kidneys of the *db/db* mice.

The IHC analysis revealed that ELA was primarily localized within renal tubular epithelial cells, with minimal staining observed in glomeruli (***Fig. 1A*** and ***1B***). A significant reduction in ELA protein levels was detected in the kidneys of the *db/db* mice (***Fig. 1C*** and ***1D***). These results suggest that the downregulation of ELA expression in renal tubular epithelial cells may be involved in the development of DKD.

### Effects of ELA on physiological and metabolic parameters in the *db/db* mice

**Figure 2:**
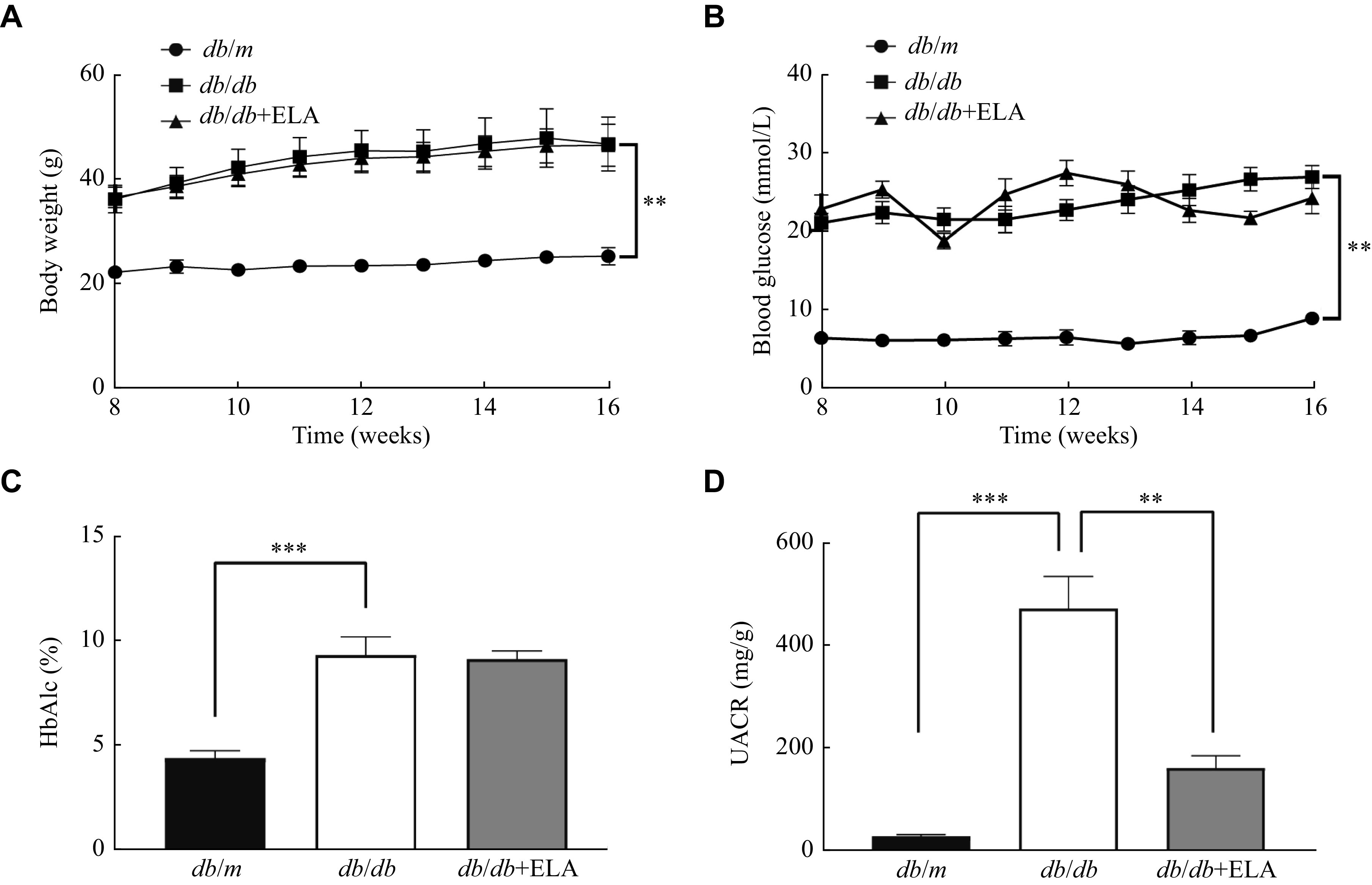
Effects of ELA on physiological and metabolic parameters in the *db/db* mice.

To investigate the potential involvement of ELA in DKD, we first administered ELA peptide to the *db/db* mice, and then measured the body weight, blood glucose, and HbA1c levels in the treated mice. We found no significant changes in these parameters following the ELA treatment (***Fig. 2A***–***2C***). At the end of the study, UACR levels were found to be elevated in the *db/db* mice; however, the ELA administration reduced urinary protein excretion (***Fig. 2D***). Collectively, these data indicate that the reno-protective effects of ELA may be independent of body weight and blood glucose regulation at the treatment doses.

### ELA attenuated renal pathological damage and fibrosis in the *db/db* mice

**Figure 3:**
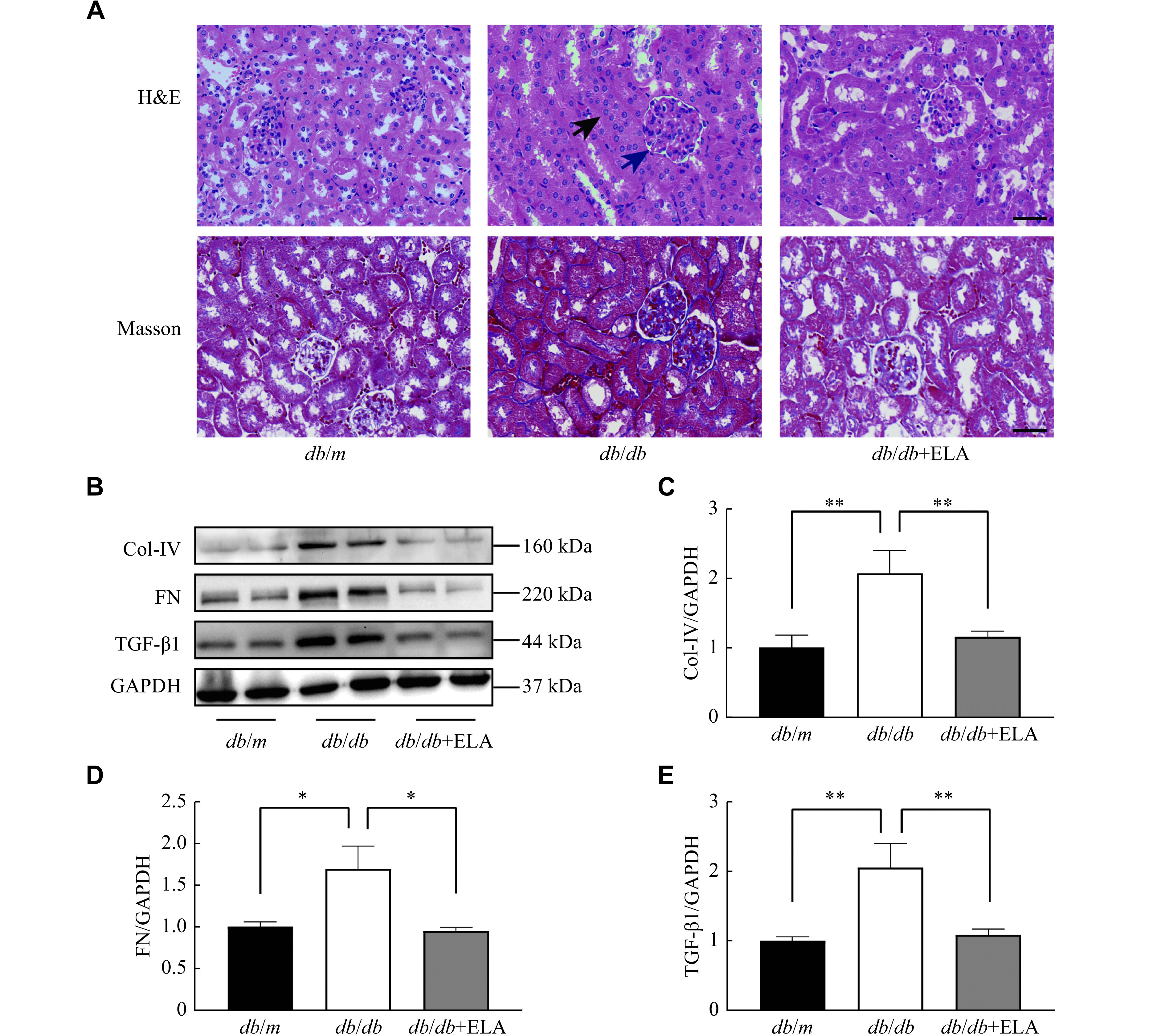
ELA attenuated renal pathological damage and fibrosis in the *db/db* mice.

We then examined general morphological changes in the kidneys using light microscopy. H&E staining revealed a significantly increased glomerular volume, tubular epithelial edema, and lumen stenosis in the *db/db* group; however, the ELA administration remarkably alleviated these pathological changes in the kidneys of the *db/db* mice. Additionally, Masson staining demonstrated a reduction in collagen deposition in both glomerular and tubulointerstitial compartments following ELA supplementation in the *db/db* mice (***Fig. 3A***), indicating that ELA protected against the development of overt fibrosis. Consistent with these observations, the ELA treatment significantly inhibited the upregulated expression of renal fibrosis-associated proteins, such as Col-Ⅳ, FN, and TGF-β1 in the *db/db* mice (***Fig. 3B***–***3E***). The above results indicated that exogenous ELA peptide antagonized diabetes-related renal damage and fibrosis.

### ELA increased Beclin-1 and LC3-Ⅱ/LC3-Ⅰ ratio expression in the *db/db* mice

**Figure 4:**
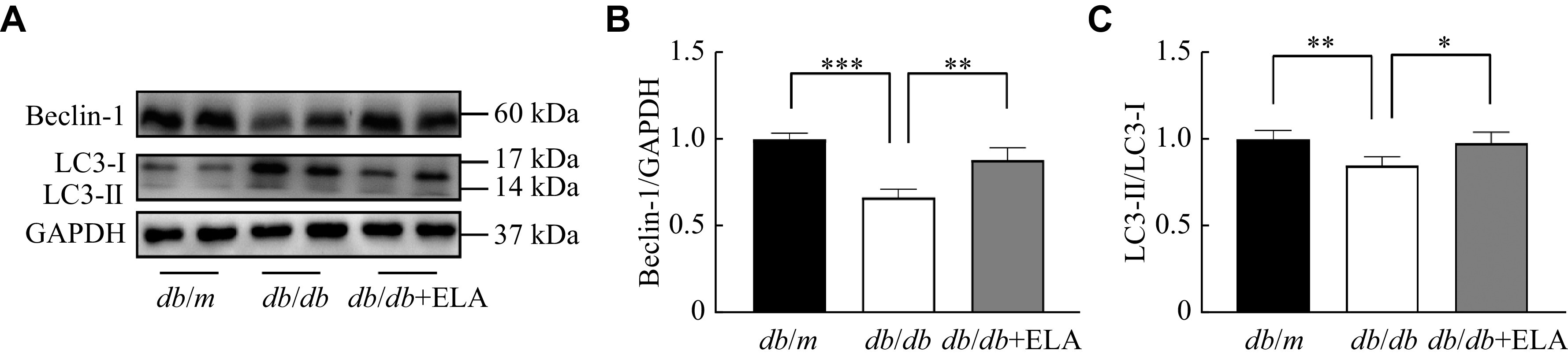
ELA increased Beclin-1 and LC3-Ⅱ/LC3-Ⅰ ratio expression in the *db/db* mice.

Given the critical role of renal tubular autophagy in DKD, we assessed the levels of autophagy-related proteins, including Beclin-1 and LC3B in mice. Beclin-1 levels and LC3-Ⅱ/LC3-Ⅰ ratios were notably diminished in the kidneys of the *db/db* mice, compared with the *db/m* mice, but significantly increased by the ELA treatment (***Fig. 4***). Taken together, these data demonstrated that ELA treatment upregulated autophagy activity under diabetic conditions.

### ELA regulated glucose-associated fibrosis and autophagy in HK-2 cells

**Figure 5:**
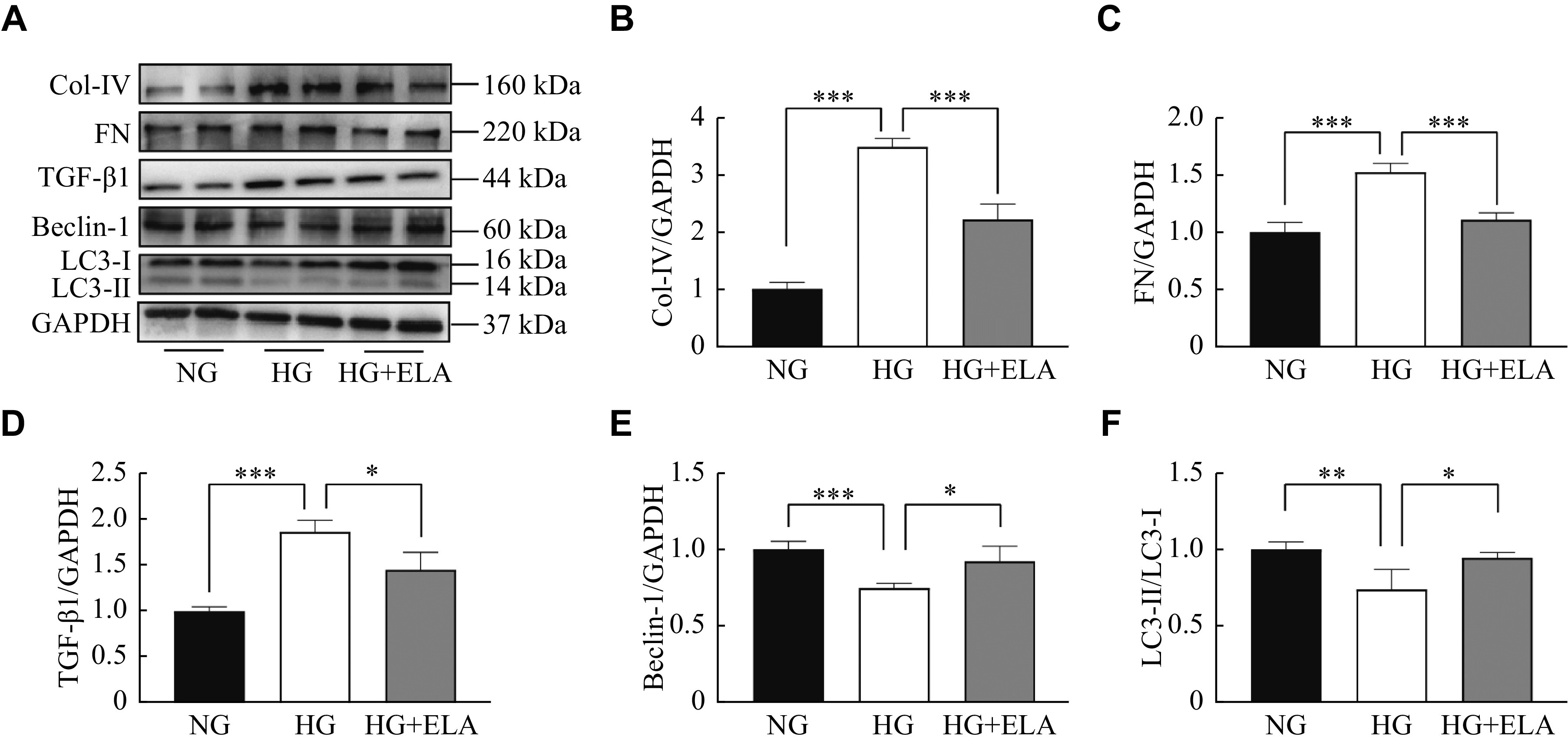
ELA regulated glucose-associated fibrosis and autophagy in HK-2 cells.

To determine the protective effects of ELA *in vitro*, we examined the expression of fibrotic factors and autophagy factors in HK-2 cells. As anticipated, Col-Ⅳ, FN, and TGF-β1 levels were elevated in a high glucose environment, while the ELA treatment blocked this elevation, signifying that ELA mitigated the fibrotic effects induced by high glucose (***Fig. 5A***–***5D***). In addition, Beclin-1 levels and LC3-Ⅱ/LC3-Ⅰ ratios were also suppressed in high glucose-treated HK-2 cells, whereas ELA significantly increased the expression of these markers (***Fig. 5A***, ***5E*** and ***5F***). These findings implied that tubular autophagy activity was inhibited by high glucose, but ELA reduced high glucose-induced ECM secretion and enhanced renal tubular autophagy.

### ELA protected against high glucose-induced injury by activation of renal tubular autophagy

**Figure 6:**
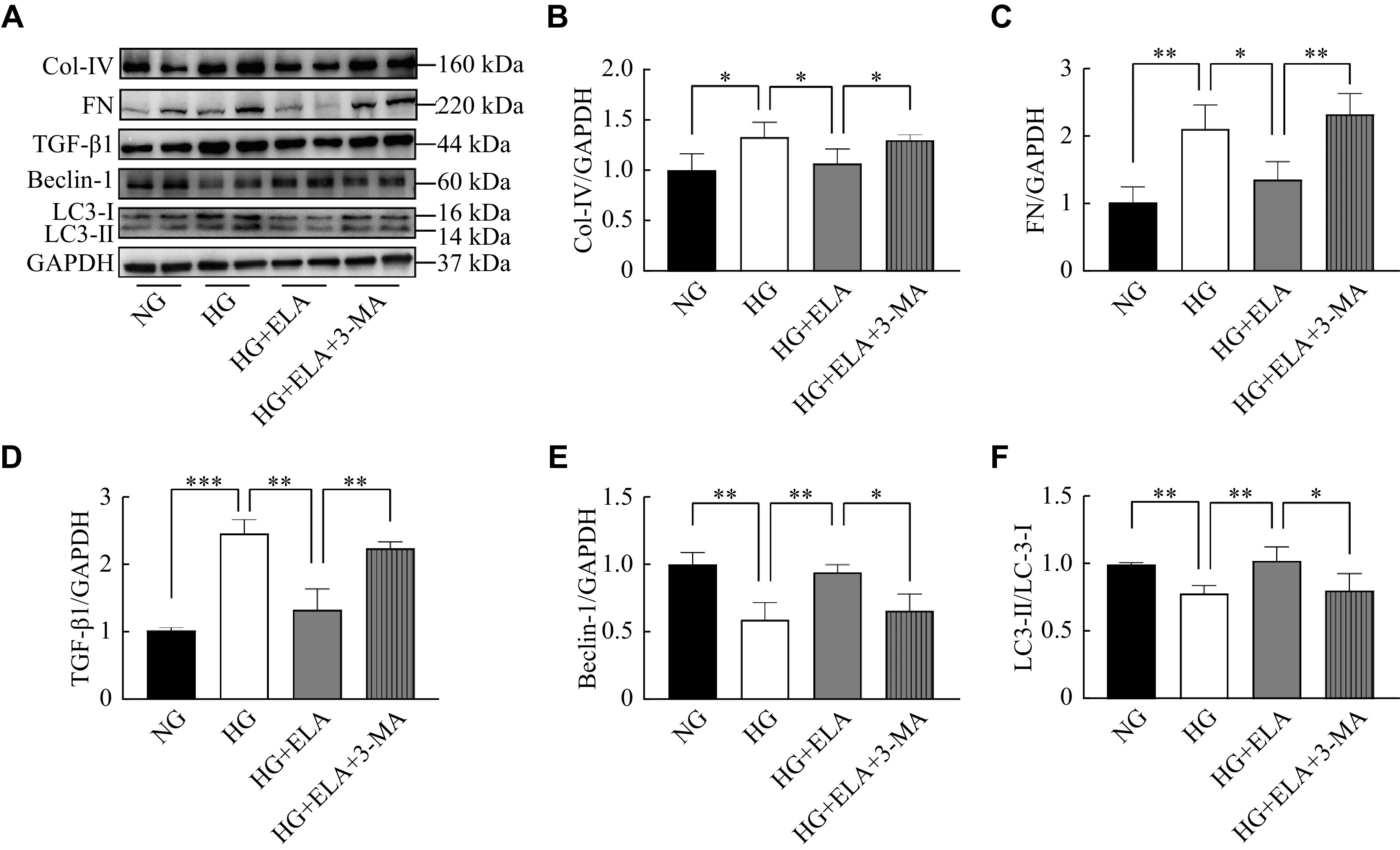
ELA protected against high glucose-induced injury by activating renal tubular autophagy.

To further investigate the mechanism of ELA involved in the improvement of DKD, 3-MA, an autophagy inhibitor, was used to estimate whether ELA was dependent on autophagy activation to reduce ECM formation. The ELA intervention remarkably suppressed the elevation of Col-Ⅳ, FN, and TGF-β1 in HK-2 cells exposed to high glucose, but this suppression effect of ELA was reversed by 3-MA (***Fig. 6A***–***6D***). Then, we examined the expression of Beclin-1 and the LC3-Ⅱ/LC3-Ⅰ ratios in high glucose-cultured HK-2 cells. Strikingly, ELA considerably increased the high glucose-associated downregulation of Beclin-1 and LC3-Ⅱ/LC3-Ⅰ ratios in HK-2 cells; however, 3-MA partially counteracted its influences (***Fig. 6A***, ***6E***, and ***6F***). Based on the above observations, ELA may alleviate renal injuries by activating high glucose-inhibited renal tubular autophagy.

## Discussion

DKD is a progressive disease that ultimately leads to renal failure and a high mortality rate^[2–3,5–6]^. Considering the limited treatment options currently available for DKD, it is crucial to explore its pathogenesis and identify new therapeutic targets^[9–11,30]^. In the current study, we uncovered that the reduced ELA expression in renal tubules had an obvious effect on DKD, and demonstrated that the ELA administration could ameliorate renal dysfunction and fibrosis. Mechanistically, ELA may exert potent renal protective effects in DKD by activating high glucose-inhibited renal tubular autophagy activity.

ELA, a novel endogenous molecule, is predominantly expressed in the kidney, heart, blood vessels, and other tissues^[24]^. It has been identified to play important roles in various organisms and has the potential to alleviate cardiac and renal remodeling^[25–26,31]^. Previous studies showed the possibility that ELA might exert beneficial effects on ischemia-reperfusion renal injury recovery^[28]^. Furthermore, ELA has been reported to exhibit an anti-fibrotic effect in mice with streptozocin-induced diabetes by inhibiting podocyte injury^[32]^. However, the protective effect of ELA on diabetic renal tubular injury has rarely been explored. In our prior studies, we found that the plasma ELA level was decreased in DKD patients^[29]^. In the current study, ELA expression was found to be reduced in renal tubular epithelial cells in the *db/db* mice. Based on this finding, we further investigated the effect of ELA on DKD, particularly on renal tubules, and sought to reveal its potential mechanism.

DKD is a clinical syndrome characterized by the increased urinary protein excretion rate and progressive loss of kidney function, which manifests as an accumulation of ECM and kidney fibrosis^[33]^. When we evaluated the role of ELA in diabetic kidney injury, we observed that the ELA-injected *db/db* mice exhibited the decreased UACR levels, compared with the *db/db* group, along with the attenuated renal fibrosis. Additionally, we also demonstrated that ELA suppressed the production of Col-Ⅳ, FN, and TGF-β1 in the diabetic cell model. Therefore, our findings indicate that ELA may possess the capacity to protect against diabetic renal damage.

An important finding of the current study is the identification of ELA as an inducer of tubular cell autophagy, functioning as a pro-survival mechanism. Autophagy serves as an adaptive response to metabolic stress, facilitating the removal of harmful constituents and recycling of cellular components to maintain cellular homeostasis^[34-35]^. Accumulating evidence shows that renal tubular autophagy is dysregulated in DKD^[18–19,21]^. Specifically, the renal tubule autophagy impairment is a key feature of DKD, contributing to renal hypertrophy and the progression of DKD. Thus, it is reasonable to propose that ELA-related renal protective functions operate, at least in part, through targeting renal tubular autophagy. This encourages further investigation into whether ELA ameliorates diabetic renal fibrosis by regulating renal tubular autophagy. Beclin-1 is a homologous gene of yeast autophagy gene *Atg6/Vps30*, an essential molecule for autophagosome formation^[36]^. LC3 controls autophagosome elongation and is an established autophagosome marker protein in mammalian cells^[37]^. During the process of autophagy, LC3-Ⅰ is converted to LC3-Ⅱ by conjugating with phosphatidylethanolamine, with the LC3-Ⅱ/LC3-Ⅰ ratio serving as a quantitative index of autophagy activity^[18]^. Our current study demonstrated the increased Beclin-1 expression and LC3-Ⅱ/LC3-Ⅰ ratio in the ELA-treated *db/db* mice, compared with the untreated *db/db* mice. To further test this hypothesis *in vitro*, we measured autophagy levels in HK-2 cells exposed to high glucose. The results revealed that high glucose markedly decreased the expression of Beclin-1 and LC3-Ⅱ/LC3-Ⅰ ratio, while ELA intervention effectively counteracted autophagy downregulation partially. Notably, ELA exhibited both anti-fibrotic and pro-autophagic effects. However, the opposing trends in fibrosis and autophagy suggest potential connections between ELA and pathological processes of renal tubular epithelial cells cultured in high glucose. Intriguingly, we observed the reduced autophagic activity and the increased ECM secretion in HK-2 cells treated with 3-MA, confirmed by the decreased Beclin-1 and LC3-Ⅱ/LC3-Ⅰ ratio. Taken together, our data suggest that ELA may alleviate ECM deposition in DKD, potentially through up-regulation of renal tubular autophagic activity.

To the best of our knowledge, this is the first study investigating the ELA peptide and its protective effects against DKD. In line with previous studies^[28,32,38]^, our current study demonstrated ELA's ameliorative effect on renal fibrosis. Moreover, we discovered that ELA protected renal tubular cells by enhancing autophagy. Up-regulation of renal tubular autophagy is beneficial to promote the elimination of ECM in the kidney, thus providing a new approach to mitigating excessive ECM accumulation through ELA-induced autophagy improvement.

However, the current study has some limitations. Firstly, we exclusively selected male mice, leaving the effect of ELA on female mice unknown. It is worth noting that ELA ameliorated renal adverse effects of eclampsia and delayed the deterioration of renal function^[27]^, suggesting the potential for similar positive effects in female mice with DKD. Future investigations should thoroughly evaluate the sex-specific effects of ELA on DKD. Secondly, the underlying mechanism of ELA up-regulating renal tubular autophagy remains insufficiently elucidated. Several signaling pathways, such as the mammalian target of rapamycin (mTOR), AMP-activated protein kinase (AMPK), and the low-energy sensors sirtuin-1 (SIRT1) pathways, have been reported to regulate autophagy^[39–41]^. Future studies are necessary to determine the specific signal pathways involved in autophagy activation. In conclusion, our findings provide some evidence that ELA may be a novel therapeutic candidate for DKD.
